# Characteristic gene expression in the liver monocyte-macrophage-DC system is associated with the progression of fibrosis in NASH

**DOI:** 10.3389/fimmu.2023.1098056

**Published:** 2023-02-24

**Authors:** Xiaoxiao Wang, Zilong Wang, Baiyi Liu, Rui Jin, Yuyun Song, Ran Fei, Xu Cong, Rui Huang, Xiaohe Li, Jia Yang, Lai Wei, Huiying Rao, Feng Liu

**Affiliations:** ^1^ Peking University People’s Hospital, Peking University Hepatology Institute, Beijing Key Laboratory of Hepatitis C and Immunotherapy for Liver Diseases, Beijing International Cooperation Base for Science and Technology on NAFLD Diagnosis, Beijing, China; ^2^ Beijing Tsinghua Changgung Hospital, Tsinghua University, Beijing, China

**Keywords:** nonalcoholic steatohepatitis (NASH), fibrosis, single cell, monocyte-macrophage-DC (MMD) system, Fmnl1, Myh9

## Abstract

**Background:**

The monocyte-macrophage-dendritic cell (DC) (MMD) system exerts crucial functions that may modulate fibrogenesis in nonalcoholic steatohepatitis (NASH). In this study, we explored the cell characteristics, distribution and developmental trajectory of the liver MMD system in NASH mice with fibrosis and clarified characteristic genes of the MMD system involved in liver fibrosis progression in NASH mice and patients.

**Methods:**

Single cells in liver tissue samples from NASH and normal mice were quantified using single-cell RNA sequencing (scRNA-seq) analysis. Differentially expressed genes (DEGs) in the MMD system by pseudotime analysis were validated by tyramide signal amplification (TSA)-immunohistochemical staining (IHC) and analyzed by second harmonic generation (SHG)/two-photon excitation fluorescence (TPEF).

**Results:**

Compared with control mice, there were increased numbers of monocytes, Kupffer cells, and DCs in two NASH mouse models. From the transcriptional profiles of these single cells, we identified 8 monocyte subsets (Mono1-Mono8) with different molecular and functional properties. Furthermore, the pseudotime analysis showed that Mono5 and Mono6 were at the beginning of the trajectory path, whereas Mono2, Mono4, Kupffer cells and DCs were at a terminal state. Genes related to liver collagen production were at the late stage of this trajectory path. DEGs analysis revealed that the genes Fmnl1 and Myh9 in the MMD system were gradually upregulated during the trajectory. By TSA-IHC, the Fmnl1 and Myh9 expression levels were increased and associated with collagen production and fibrosis stage in NASH mice and patients.

**Conclusions:**

Our transcriptome data provide a novel landscape of the MMD system that is involved in advanced NASH disease status. Fmnl1 and Myh9 expression in the MMD system was associated with the progression of NASH fibrosis.

## 1 Introduction

Nonalcoholic fatty liver disease (NAFLD) is defined as excessive fat deposition in the liver and in absence of heavy drinking and other chronic liver diseases (1, 2). The natural history of NAFLD consists of steatosis, nonalcoholic steatohepatitis (NASH), fibrosis, cirrhosis and hepatocellular carcinoma (HCC) (1–3). Approximately one-quarter of the general population has NAFLD, 10-30% of NAFLD patients are at risk of progressing to NASH and liver fibrosis, and 0.3-3% of NASH patients with fibrosis progress to cirrhosis and HCC annually (4). In particular, fibrosis is considered as a crucial adverse result in the natural process of NASH progression.

Fibrosis is independently associated with liver transplantation and liver-related risk events in patients with NASH (5–7). The relative risks of hazardous events increased from fibrosis stage 2, especially in NASH patients with cirrhosis, the occurrence of liver decompensation and the mortality of liver-related events and all-cause mortality increased substantially (5, 8, 9). Therefore, growing emphasis has been placed on improving or treating fibrosis in NASH (10). However, there are currently no approved drugs for NAFLD and NASH by the Food and Drug Administration (FDA), especially for NASH fibrosis.

At present, the mechanism of liver fibrogenesis in NASH is unclear. Previous studies have shown that monocytes, macrophages and dendritic cells (DCs) exert diversity in modulating fibrogenesis (11, 12). In the healthy state, the majority of macrophages are liver-resident yolk sac-derived Kupffer cells (13), while in the setting of liver injury, including NASH, there is marked infiltration of monocyte-derived macrophages (MoMFs) (14). Furthermore, under inflammatory settings, monocytes can also differentiate into a special subset of DCs, called moDCs (monocyte-derived DCs) (15). However, until recently, the characteristic genes expressed in the monocyte-macrophage-DC (MMD) system that are involved in the progression of NASH fibrosis were not fully understood. In recent years, single-cell transcriptomics provides high-dimensional information about cellular composition of various tissues, which reshapes and updates the understanding of complex biological systems for researchers (16). Although single-cell RNA sequencing (scRNA-seq) has been conducted and applied in the livers of mice and humans, few studies have investigated the characteristics of the MMD system in the fibrogenesis of NASH. In this study, we explored the cell composition and developmental trajectory of cells in the MMD system and clarified the relationship between hub genes from the MMD system and collagen deposition in NASH fibrosis.

## 2 Material and methods

### 2.1 Experimental mouse models

C57BL/6 mice (RRID: MGI:2159769) were fed a high-fat diet (HFD, 23.6% fat, 41.5% carbohydrate and 0.02% cholesterol; MD12032, Medicience Ltd., China) or western diet (WD, 21.2% fat, 48.5% carbohydrate, and 1.25% cholesterol; TD120528, Medicience Ltd., China) and fructose solution (23.1 g fructose and 18.9 g glucose in a liter of tap water) plus intraperitoneal injection of CCl4 (carbon tetrachloride, 10%, 2.5ul/g body weight, once a week) (HFD+F+CCl4 and WD+F+CCl4) for 16 weeks. In addition, the control mice were given normal diet (18% kcal fat, 58% kcal carbohydrate, SPF Biotechnology Co., Ltd.) and drinking water. Before model construction, mice were assigned into control and two experimental groups using randomizations. After 16 weeks, no mice were excluded from our study and all eight mice are available for final analysis. This study was approved by the Ethics Committee of Peking University People’s Hospital (2021PHE111) and conformed to the ethical guidelines of the 1975 Declaration of Helsinki.

### 2.2 Tissue dissociation, preparation of single-cell suspensions, Chromium 10x Genomics library and sequencing

Liver tissues of mice were obtained from wild-type mice (N=2) and NASH mice with fibrosis (HFD+F+CCl4, N=3; WD+F+CCl4, N=3) after heart perfusion. Subsequently, liver tissue of each mice was transferred into a sterile RNase-free culture dish containing 1x PBS (calcium and magnesium-free) on ice and cut it into 0.5 mm^2^ pieces. Liver tissues were dissociated into single cells in dissociation solution (0.35% collagenase IV5, 2 mg/ml papain, 120 Units/ml DNase I) in 37 °C water bath with shaking for 20 min at 100 rpm. The cell suspension was filtered by passing through 70-30 um stacked cell strainer and centrifuged at 300g for 5 min at 4°C. The cell pellet was resuspended in 100 ul 1x PBS and added with 1 ml 1x red blood cell lysis buffer (MACS 130-094-183) and incubated at room temperature for 2-10 min to lyse remaining red blood cells. After incubation, the suspension was centrifuged at 300g for 5 min. The suspension was resuspended in 100μl Dead Cell Removal MicroBeads (MACS 130-090-101) and remove dead cells using Miltenyi^®^ Dead Cell Removal Kit (MACS 130-090-101). Then the suspension was resuspended in 1 xPBS and centrifuged at 300 g for 3 min. The cell pellet was resuspended in 50 μl of 1 x PBS. The overall cell viability was confirmed by trypan blue exclusion, which needed to be above 85%.

Depending on the manufacturer’s instructions of the 10X Genomics Chromium Single-Cell 3’ Kit (V3), single-cell suspensions were loaded into 10x Chromium. Then, according to the standard operation protocols, we performed cDNA amplification and library construction. Libraries were sequenced on an Illumina NovaSeq 6000 sequencing system (paired-end multiplexing run, 150 bp) at a minimum depth of 20,000 reads per cell by LC-BioTechnology Co., Ltd. (HangZhou, China).

### 2.3 Single-cell RNA sequencing data analysis

Using Illumina bcl2fastq software (version 2.20), sequencing results were demultiplexed and converted to FASTQ format. Sample demultiplexing, barcode processing and single-cell 3’ gene counting were performed depending on the Cell Ranger pipeline (version 5.0.1, https://support.10xgenomics.com/single-cell-geneexpression/software/pipelines/latest/what-is-cell-ranger), and scRNA-seq data were aligned to the Ensembl genome GRCh38/GRCm38 reference genome. About 88,000 single cells captured from 8 mice were processed using 10X Genomics Chromium Single Cell 3’ Solution. The Cell Ranger output was loaded into Seurat (version 3.1.1) and used for dimensional reduction, clustering, and scRNA-seq data analysis.

### 2.4 Cell annotation and comparison of differentially expressed genes

We completed our manual clusters annotations depending on marker genes of specific cells from published papers and CellMarker (CellMarker/index.jsp). The key differentially expressed transcripts that define each cell cluster are exhibited in **Table 1**. We performed bioinformatic analysis using the OmicStudio tools at https://www.omicstudio.cn/tool.

**Table 1:** The representative markers of liver cells for single cell analysis.

Cell	Marker
Hepatocyte	Afp, Alb
Hepatic stellate cell	Acta 1, Des
Endothelial cell	Bmp2, Lyvel
Dendritic cell	Itgax, CD83, CD80
monocyte	Ly6c1, Ly6c2 and Itgam
Kupffer cell	Clec4f, Adgre1
T cell	CD3d, CD3e, CD3g, CD4, CD8a
B cell	CD22
granulocyte	Itgam and Ly6g

### 2.5 Cell developmental trajectory analysis

Using Monocle2, we inferred the cell lineage trajectory of the MMD system. We first used the transcript count data (e.g. UMI) and created an object with the parameter ‘‘expression Family = negbinomial. Size ()’’ following the Monocle2 tutorial. The ‘‘differentialGeneTest’’ function was used to derive differentially expressed genes (DEGs) from each cluster, and genes with a q-value < 0.05 were used to order the cells in pseudotime analysis. Furthermore, DEGs along the pseudotime were detected and analyzed using the ‘‘differentialGeneTest’’ function after the cell trajectories were constructed.

### 2.6 Preparation and pathological evaluation of human and mouse liver slices

Human paraffin-embedded liver tissues were obtained from the Department of Pathology at Peking University People’s Hospital. In this study, 6 liver tissue samples from biopsy-proven NASH patients with fibrosis and 3 samples from healthy donors (age ≥18 years) were eligible and included. Patients with viral hepatitis, alcoholic liver disease, drug-induced liver disease, autoimmune liver disease, cholestatic liver disease or hereditary metabolic liver disease were not ineligible. We did not assess whether subjects were male or female because sex was not the analysis object in this study. We did not check for sample sizes using a power analysis because our study does not report statistics on between groups or within group variables. Informed consent forms were obtained from all participants. This study was approved by the Ethics Committee of Peking University People’s Hospital (2022PHB088-001) and conformed to the ethical guidelines of the 1975 Declaration of Helsinki.

Each sample (human and mouse) was sectioned at a thickness of 4 μm for histological assessment and immunohistochemistry (IHC). Using hematoxylin and eosin (H&E) and Sirius Red (SR) staining, liver histology for all participants was evaluated by two specialized pathologists who were blinded to the patient and mouse details according to the NASH Clinical Research Network (NASH CRN) System (steatosis was scored from 0-3, ballooning: 0-2, lobular inflammation: 0-3, portal inflammation: 0-2, and liver fibrosis: 0 to 4).

### 2.7 Second harmonic generation/two-photon excitation fluorescence

Images of unstained sections of liver tissues were acquired using a Genesis system (HistoIndex Pte. Ltd., Singapore). Collagen in liver tissues was visualized by SHG microscopy, and TPEF microscopy was used to visualize the other cell structures.

### 2.8 Multiplexed immunohistochemical staining

Multiplex staining of paraffin-embedded liver tissues was performed using a PANO 7-plex IHC kit (Cat# 0004100100, Panovue). Fmnl1 (1:200, Novus Cat# NBP1-88460, RRID: AB_11040849), Myh9 (1;3000, Abcam Cat# ab238131, RRID: AB_2924880), CD11c (1:150, CST Cat# 97585, RRID: AB_2800282), Ly6C (1:50, Abcam Cat# ab54223, RRID: AB_881384), CLEC4F (1:50, R and D System Cat# MAB2784, RRID: AB_2081338), CD11c (1:800, CST #45581, RRID: AB_2799286), CD68 (1:800, Abcam Cat# ab955, RRID: AB_307338) and CCR2 (1:500, Origene Cat# TA337218, RRID: AB_2924881) antibodies were sequentially applied, followed by horseradish peroxidase-conjugated secondary antibody incubation and tyramide signal amplification (TSA). After all antigens above have been labeled, nuclei were stained with 4’-6’-diamidino-2-phenylindole (DAPI, Sigma-Aldrich, Missouri, USA, Cat# D9542). The stained slides were scanned to obtain multispectral images using the Mantra System (PerkinElmer) and analyzed using InForm image analysis software (version 2.4, PerkinElmer).

### 2.9 Cell culture, detection, and analysis

On the one hand, THP1 cells were seeded 1x10^6^ cells per well in six-well plate and treated with palmitate (PA,1 mM, Sigma-Aldrich, USA). Meanwhile, different siRNA-lipofectamine™ 3000 (Cat# L3000015, Invitogen) mixture was added into cell culture medium. After 48h, total RNA of THP1 cells were extracted to detect the levels of Jun, spp1, Socs3 and Rac1. On the other hand, THP1 cells were seeded 1x10^6^ cells per well on six-well upper trans-well insert (0.4µM), LX2 cells were seeded 1x10^5^ cells in the lower wells of trans-well plates and attached overnight. After 24h, THP1 cells were washed with phosphate-buffered saline solution (PBS), and treated with PA (1 mM) and different siRNA-lipofectamine™ 3000 mixture, each upper insert was transferred to a lower plate containing the LX2 cells. Thereafter, THP1 cells and LX2 cells were co-cultured in serum-free medium for 48 hours. Total RNA of LX2 cells were extracted to detect the levels of α-smooth muscle actin (SMA), collagen type I alpha 1 (Col1a1) and Fibronectin (Fn). The sequences of different siRNA were listed in **Supplementary Table 1**.

### 2.10 Statistical analysis

Statistical analysis was performed using R 4.1.2 (Vienna, Austria), SPSS 20.0 (SPSS, Chicago, IL, United States) and Graphpad Prism 5.0 (Graphpad Software Inc., San Diego, CA, United States). Comparisons of cell distribution and gene expression among different groups were performed using one-way ANOVA. Comparisons of mRNA levels between two coculture groups were analyzed using the Mann−Whitney U test. Two-sided p-values less than 0.05 were considered significant.

## 3 Results

### 3.1 NASH mice showed obvious liver steatosis, inflammation, and fibrosis

**Figure 1:**
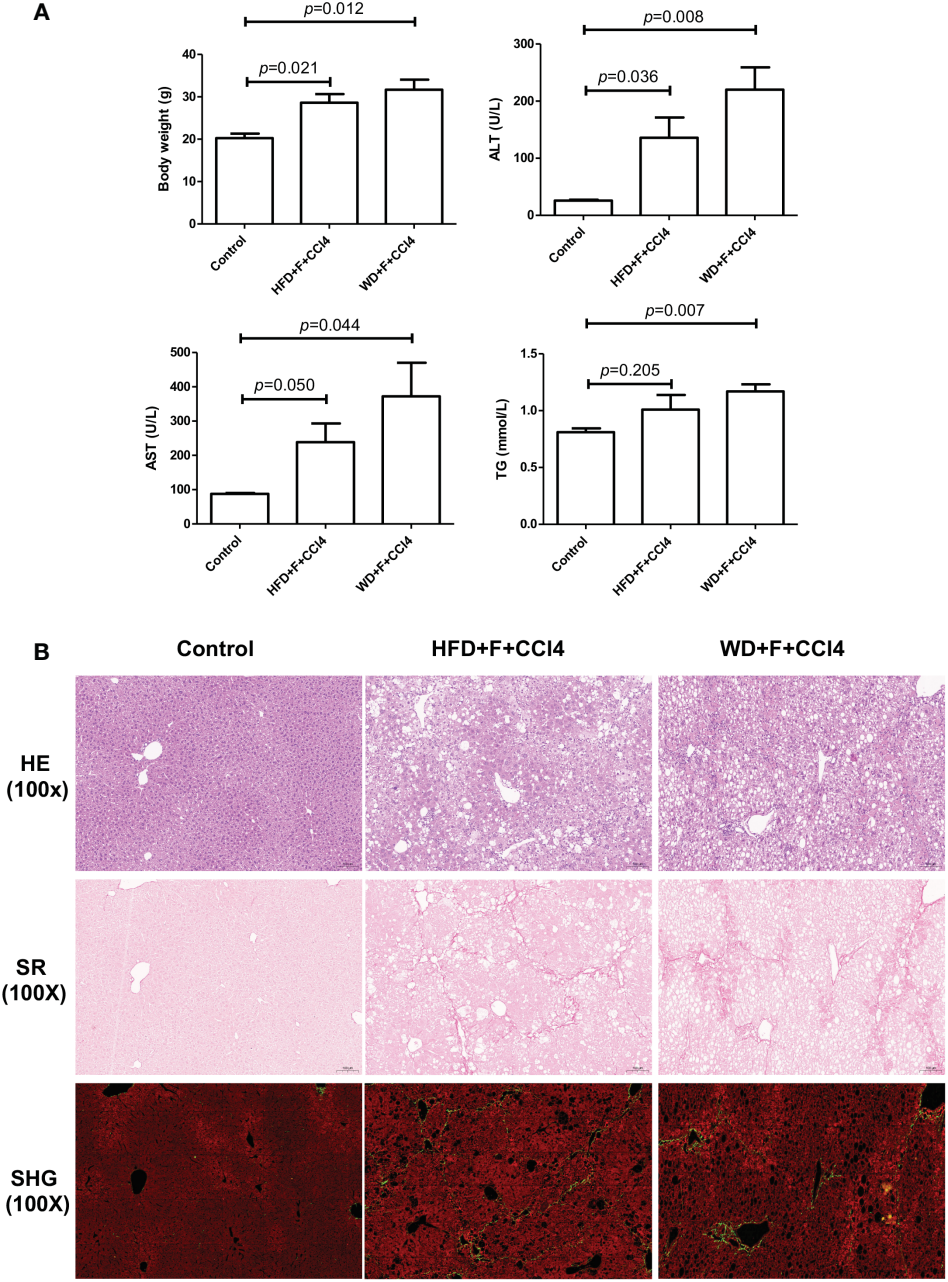
Metabolic and pathological characteristics of NASH mice with fibrosis. **(A)** Body weight, ALT, AST and TG levels and **(B)** Hematoxylin and eosin (H&E), Sirius Red staining and SHG/TPEF scanning images in control, HFD+F+CCl4 and WD+F+CCl4 mice. ps: ALT, alanine aminotransferase; AST, aspartate aminotransferase; and TG, triglyceride.

Compared with mice fed a normal diet, two NASH mouse models with fibrosis developed pronounced obesity. In response to HFD/WD feeding, fructose water and CCl4, NASH mice showed significantly increased body weight and serum alanine aminotransferase (ALT), aspartate aminotransferase (AST) and triglyceride (TG) levels, especially in WD+F+CCl4 mice (**Figure 1A**). In addition, by HE and SR staining evaluation, there was obvious macrovesicular and microvesicular steatosis (score 2-3), lobular inflammation (score 2-3), and lobular and portal fibrosis (score 3-4) in NASH mice (**Figure 1B**) after constructed for 16 weeks. Although there was no statistical difference, we observed obvious collagen deposition and steatosis signals in images from SHG/TPEF scanning of NASH mice (**Figure 1B** and **Supplementary Figure 1**).

### 3.2 ScRNA-seq profiling of liver cells in NASH mice with fibrosis

**Figure 2:**
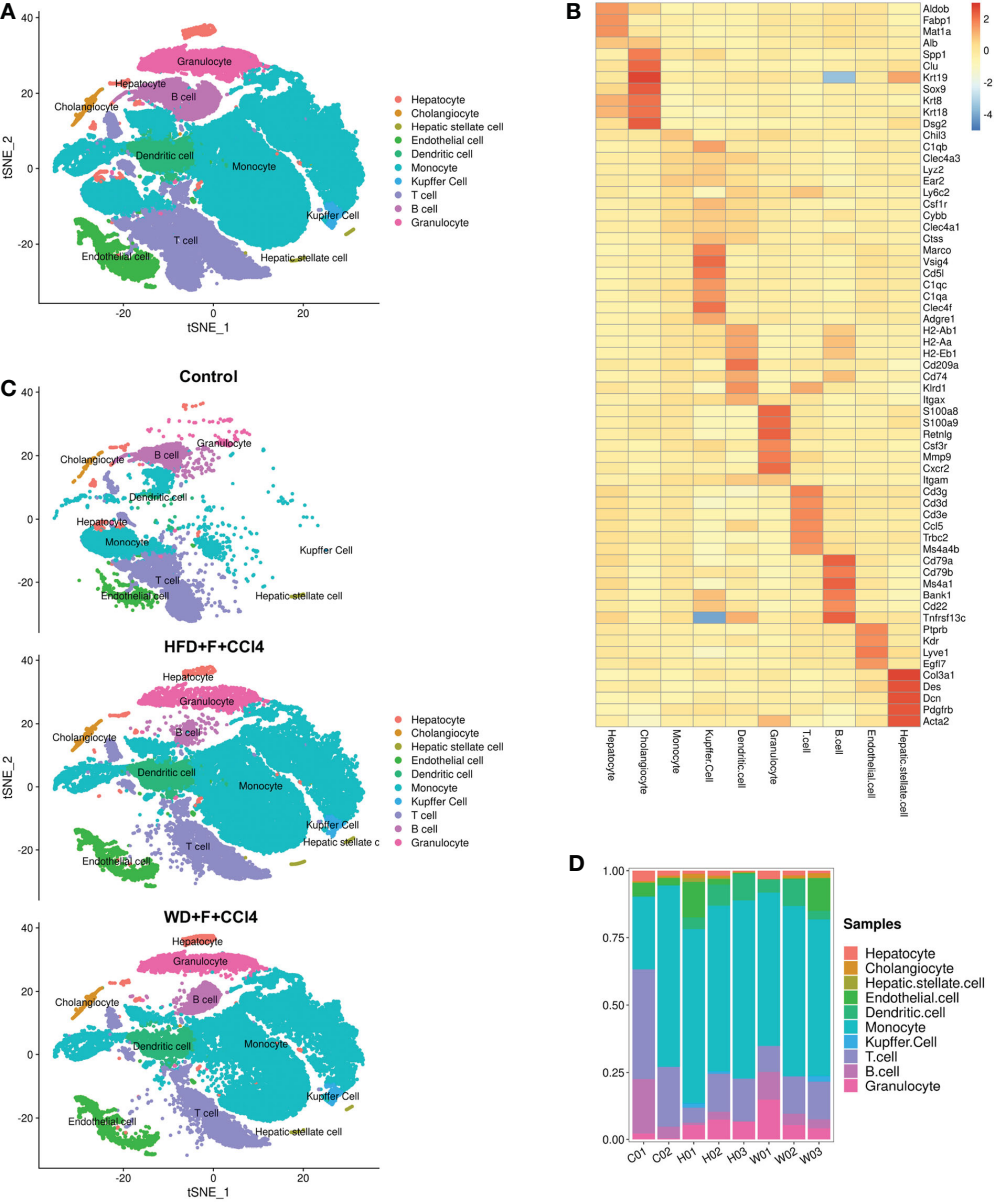
ScRNA-seq profiling of liver cells from normal and NASH mice with fibrosis. **(A)** The annotation and color codes for the 10 identified liver cells. **(B)** The t-SNE plot shows the cell origins in the control, HFD+F+CCl4 mice and WFD+F+CCl4 mice. **(C)** Heatmap showing the expression of marker genes in the indicated cell types. **(D)** Histogram indicating the proportion of cells in the liver tissue of each mouse. ps: C01-02: control mice 01-02; H01-03: HFD+F+CCl4 mice 01-03; W01-03: WFD+F+CCl4 mice 01-03.

To construct a liver cell atlas in NASH mouse models, we performed cell classification and marker gene identification. After the filtration of low-quality cells, there were 14956 cells in the control group, 31956 cells in the HFD+F+CCl4 group, and 33562 cells in the WD+F+CCl4 group. The requirements for high-quality cells in this study are as follows: 1. The number of genes identified in a single cell (500 Inf); 2. The total number of UMIs in a single cell was less than Inf; 3. The percentage of mitochondrial gene expression in a single cell was less than 25%. In addition, genes were filtered to retain genes expressed in at least one cell. A total of 27 cell clusters were identified and visualized using the T-distributed stochastic neighbor embedding (t-SNE) method in control mice and NASH mice with fibrosis (**Supplementary Figures 2A, B**). According to the representative markers of live cells in **Table 1**, we identified 10 different cells (**Figure 2A**). The parenchymal cells of the liver are mainly hepatocytes, and nonparenchymal cells consist of cholangiocytes, hepatic stellate cells (HSCs), endothelial cells, DCs, monocytes, Kupffer cells, T cells, B cells, and granulocytes (**Figures 2A, B** and **Supplementary Figure 3**). All of these cell subtypes were shared among control mice and NASH mice with fibrosis, however, at different proportions. Compared with those of control mice, higher proportions of monocytes, Kupffer cells and DCs were observed in HFD+F+CCl4 and WD+F+CCl4 mice (**Figures 2C, D**). In addition, the two NASH fibrosis models shared similar cell compositions and proportions (**Figure 2C**).

### 3.3 The components and function of the monocyte-macrophage-DC system in the livers of NASH mice with fibrosis

**Figure 3:**
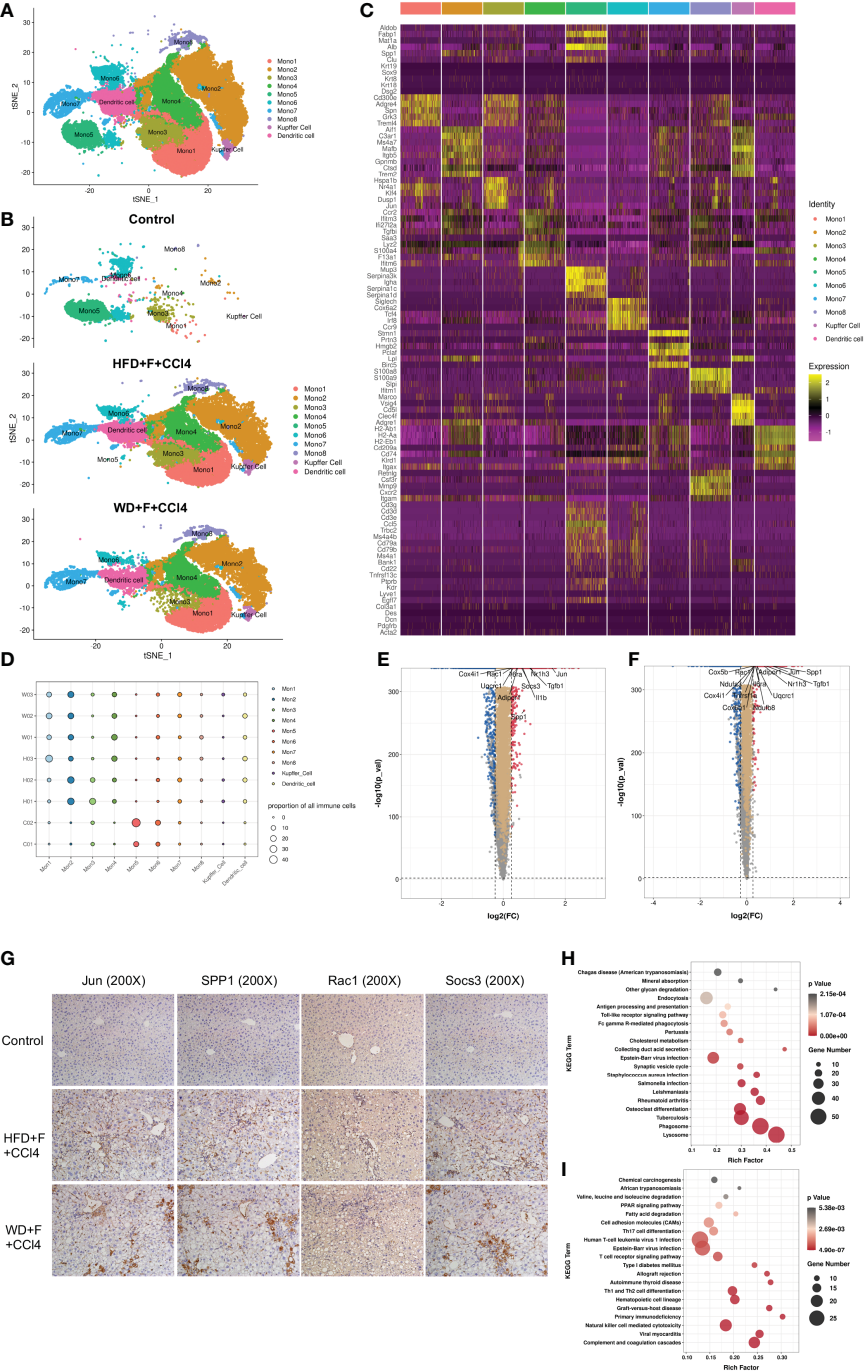
The components of the monocyte-macrophage cell-DC (MMD) system in normal and NASH mice with fibrosis. **(A, B)** t-SNE plot showing the subtypes of cells in the MMD system from normal and NASH mice with fibrosis. Each cluster is color-coded according to cell type and mouse group. **(C)** Heatmap showing the expression of the marker genes in each cell type of the MMD system. **(D)** Bubble map showing the proportion of each cell type in the MMD system from normal and NASH mice with fibrosis. **(E, F)** Volcano plot showing the differentially expressed genes in the MMD system between control and HFD+F+CCl4 mice/control and WD+F+CCl4 mice. **(G)** Representative images of IHC staining (Jun, SPP1, Rac1 and Socs3) in formalin-fixed paraffin-embedded (FFPE) tissue in control, HFD+F+CCl4 and WD+F+CCl4 mice. **(H, I)** Bubble plot showing the KEGG enrichment of specific pathways in monocyte 2 (Mono2) and Mono5. ps: C01-02: control mice 01-02; H01-03: HFD+F+CCl4 mice 01-03; W01-03: WFD+F+CCl4 mice 01-03.

We next conducted unsupervised clustering of monocytes, Kupffer cells and DCs. A total of 10 clusters emerged within the monocyte-macrophage-DC system, including eight clusters of monocytes (Mono 1-Mono 8), one cluster of Kupffer cells and one cluster of DCs (**Figures 3A–C**), namely, the MMD system. Compared with those of the control mice, there were higher proportions of Mono 1-4, Mono 7, Mono 8, Kupffer cells and DCs and lower proportions of Mono 5 and Mono 6 in the two NASH mouse models with fibrosis (**Figure 3D**). Cells in the MMD system in the two NASH fibrosis models displayed high expression levels of C-Jun, spp1, Rac1 and Socs3 (**Figures 3E, F**). IHC staining verified the increased abundance of these markers in NASH mice with fibrosis (all p<0.05, **Figure 3G** and **Supplementary Figure 4**). KEGG enrichment analysis showed increased phagosome and lysosome signaling pathways in Mono 2 and increased PPAR signaling pathways and fatty acid degradation in Mono 5 (**Figures 3H, I**). *In vitro*, Jun, Spp1, Rac1 or Socs3 in THP1 cells was knock-down (KD), and palmitic acid (PA) was used to induce lipogenesis in THP1 cells. After 48h, the mRNA levels of molecules in phagocytosis and lysosomal signal pathway (Ctsz, Psap, LAMP1) in THP1 cells decreased (**Supplementary Figure 6**).

### 3.4 The transition trajectory of cells in the MMD system of NASH mice with fibrosis

**Figure 4:**
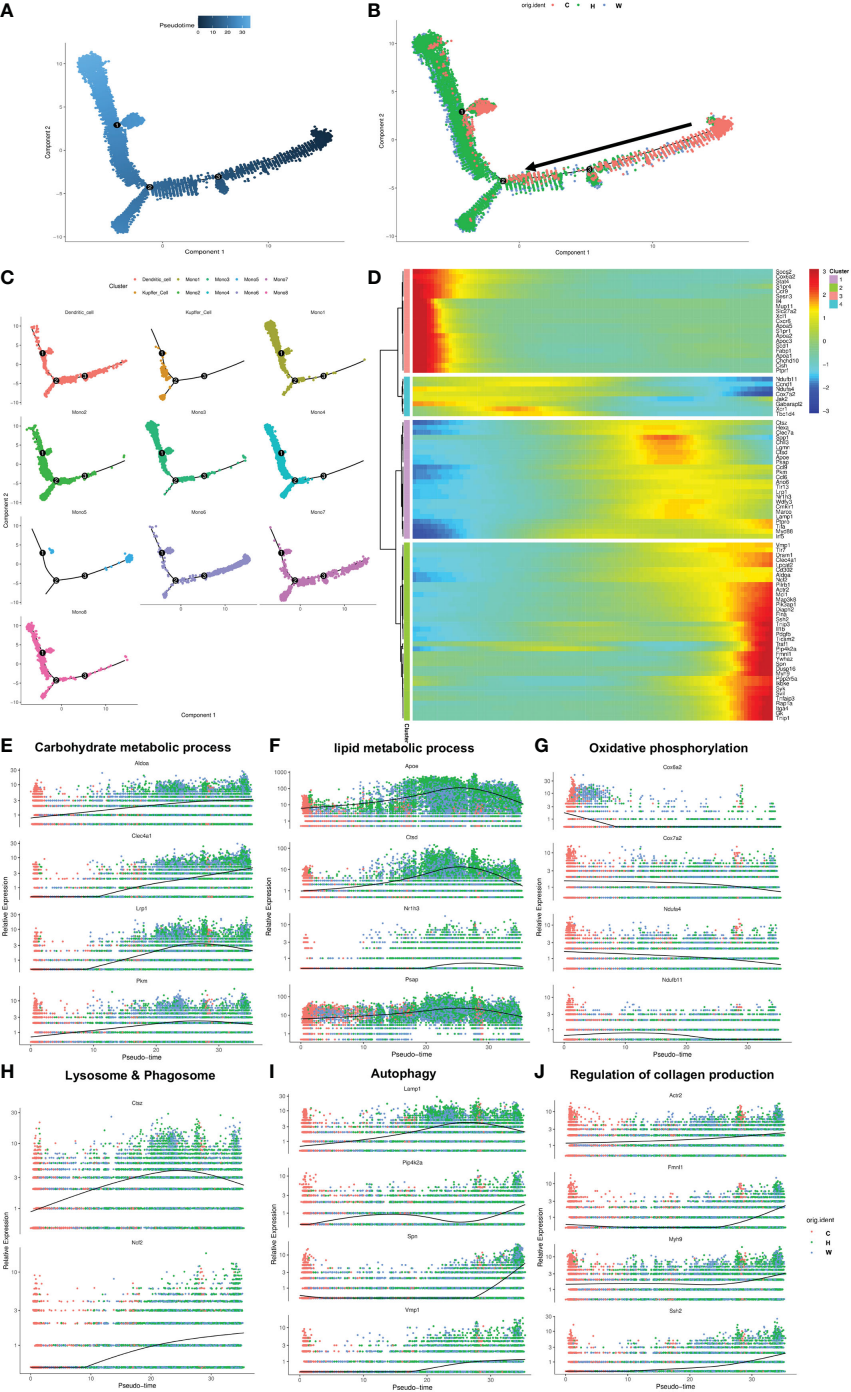
The transition trajectory and regulatory networks of cells in the MMD system. **(A, B)** Pseudotime-ordered analysis of cells in the MMD system from control and NASH mice. **(C)** Pseudotime plot showing the transition trajectory of Kupffer cells, DCs and 8 monocytes. **(D)** Heatmap showing the dynamic changes in gene expression along the pseudotime axis. **(E-J)** Characteristic genes in the select signaling pathway that are differentially expressed across the pseudotime of cells in the MMD system. ps: C, control mice; H, HFD+F+CCl4 mice; W, WFD+F+CCl4 mice.

Having defined different subsets in the MMD system, we aimed to analyze the dynamic immune states, cell transitions and pathways that further guide the different fates of cells. First, a complete dataset of monocytes, Kupffer cells and DCs was extracted and analyzed with Monocle2, which compare all single-cell transcriptomes in multidimensional space using machine-learning algorithms and orders cells along a path representing a developmental trajectory in theoretical time, known as pseudotime. Outcomes of pseudotime analysis showed that Mono5, Mono6 and Mono7 were at the beginning of the trajectory path, whereas Mono1, Mono2, Mono4, Kupffer cells and DCs were at the terminal state of the trajectory path. Surprisingly, Mono5 and Mono7 were primarily distributed in control mice, with few cells identified at the start of transition trajectory, whereas Kupffer cells and Mono1-4 were primarily distributed in the two NASH mouse models with fibrosis and mainly at the end of the transition path (**Figures 4A–C**).

According to the ordered transition trajectory, we further performed DEG analysis to explore changes in the function of pseudotime and identified four broad patterns of gene expression dynamics (**Figure 4D**). Group 1 genes were upregulated along the trajectory early and maintained continuously, and a number of factors within carbohydrate metabolic processes were enriched in this group (**Figure 4E**). Group 2 genes represented a series of genes associated with lipid metabolic processes. These genes were at higher levels at the beginning of the trajectory and then upregulated significantly and downregulated at the end of the trajectory, which was consistent with the change in liver steatosis in the progression of NASH (**Figure 4F**). Group 3 encompassed genes associated with oxidative phosphorylation, which was downregulated consistently along the trajectory (**Figure 4G**). Groups 4 and 5 were enriched for genes related to lysosome and phagosome and autophagy, which increased slightly during the trajectory (**Figures 4H, I**). Group 6 included genes associated with collagen production, which increased in expression later along the trajectory path in accordance with the progression of fibrosis in NASH (**Figure 4J**).

### 3.5 The upregulated expression of Fmnl1 and Myh9 in the MMD system is associated with collagen production in NASH mice with fibrosis

**Figure 5:**
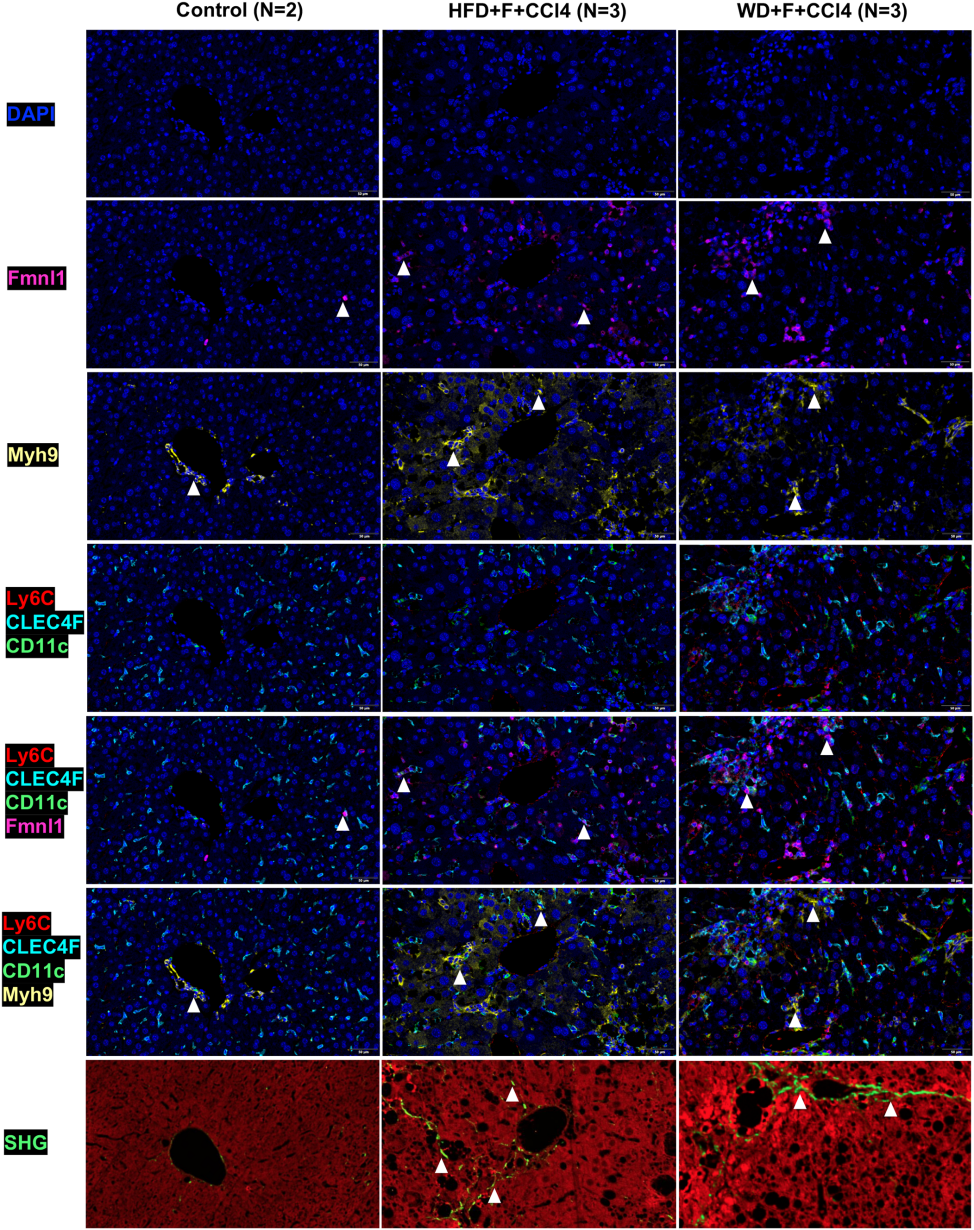
Fmnl1 and Myh9 expression in MMD system cells and collagen deposition in livers from control mice and NASH mice with fibrosis. ps: MMD: monocyte-macrophage-DC (MMD); DAPI: cell nucleus, Fmnl1: Fmnl1+ cells (white arrow), Myh9: Myh9+cells (white arrow); Ly6C/CLEC4F/CD11c/Fmnl1: Fmnl1expression in MMD system cells (white arrow); Ly6C/CLEC4F/CD11c/Myh9: Myh9 expression in MMD system cells (white arrow); SHG, collagen detected by second harmonic generation.

**Figure 6:**
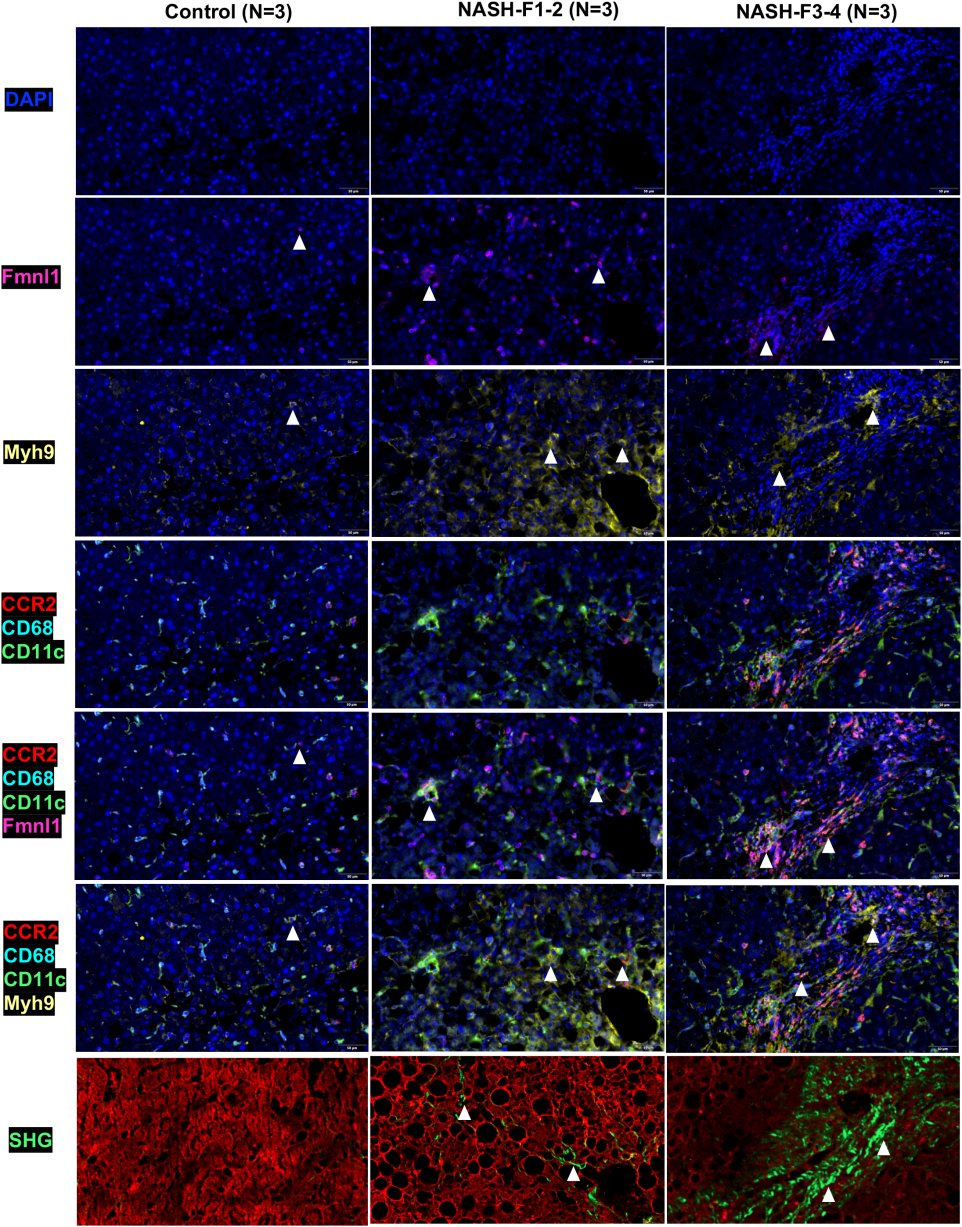
Fmnl1 and Myh9 expression in MMD system cells and collagen deposition in livers from healthy controls and NASH patients with fibrosis. ps: MMD: monocyte-macrophage-DC (MMD); F1-2: Fibrosis stage 1-2, F3-4: Fibrosis stage 3-4. DAPI: cell nucleus, Fmnl1: Fmnl1+ cells (white arrow), Myh9: Myh9+cells (white arrow); CCR2/CD68/CD11c/Fmnl1: Fmnl1 expression in MMD system cells (white arrow); CCR2/CD68/CD11c/Myh9: Myh9 expression in MMD system cells (white arrow); SHG, collagen detected by second harmonic generation.

By TSA-IHC, the expression levels of Fmnl1 and Myh9 in liver sections were increased in the two NASH mouse models with fibrosis compared with control mice. The number of MMD system cells (Ly6c/CLEC4F/CD11c+ cells) in the two NASH models was also increased (all p<0.05, **Figure 5** and **Supplementary Figure 5**). Furthermore, Fmnl1 and Myh9 were mainly expressed in the MMD system. In the livers of NASH mice with fibrosis, the expression levels of Fmnl1 and Myh9 in the MMD system were associated with the distribution of collagen detected by SHG (**Figure 5**). In the human liver, the expression levels of Fmnl1 and Myh9 in the MMD system (CCR2/CD68/CD11c+ cells in humans) were also increased with collagen deposition, and the expression levels of Fmnl1 and Myh9 increased with aggravation of the liver fibrosis stage (all p<0.05, **Figure 6** and **Supplementary Figure 5**). In order to validate the function of Fmnl1 and Myh9 in MMD system, *in vitro*, Fmnl1 or Myh9 in THP1 cells was knock-down (KD), and palmitic acid (PA) was used to induce lipogenesis of THP1 cells. Then THP1 cells were co-cultured with hepatic stellate cell line (LX2 cells). After 48h co-culture, compared with controls, in the Fmnl1 or Myh9 KD group, the mRNA levels of collagen related markers (α-SMA, Col1a1, Fibronectin) in LX2 cells were downregulated (**Supplementary Figure 7**).

## 4 Discussion

Monocytes, macrophages and DCs are crucial nonparenchymal cells in the liver, and these three immune cells have the same origin during NASH (17). In our study, they were collectively named the MMD system. During the pathogenesis of NASH, monocytes migrate from peripheral blood and transform into macrophages and/or DCs in the liver, which participate in the progression of liver fibrosis (14, 15, 17, 18). In this study, using scRNA-seq, we found that the number and distribution of monocytes, Kupffer cells and DCs changed significantly during NASH fibrosis. In the process of NASH fibrosis, some types of monocytes in the MMD system transformed into new monocytes, macrophages and DC cells, and Fmnl1 and Myh9 levels in the MMD system were also significantly increased at the end of the trajectory path, which was associated with the deposition of liver collagen in NASH mice and patients with fibrosis.

Compared with control mice, the numbers and types of monocytes, Kupffer cells and DCs in NASH mice with fibrosis were increased, especially monocytes. Similarly, by CITE-seq, researchers found that the NAFLD mouse model showed diverse macrophage subtypes (19). Seidman et al. found that there were five major macrophage subsets in the liver of NASH mice, including normal KCs, NASH KCs, recruited macrophages (RM), Ly6C^hi^-RM and Ly6C^lo^-RM (20). During liver injury, whether acute or chronic injury, a large number of monocytes are recruited to sites of hepatic injury, at this time, monocytes can differentiate into macrophages and become the main macrophage population, which promotes the activation of hepatic stellate cells (HSCs) to become myofibroblasts and contributes to NASH fibrogenesis (21–24). Furthermore, DCs in the liver also appear to be involved in liver fibrosis in the progression of NAFLD (25). During fibrosis induced by TAA and recombinant leptin, the number of DCs were elevated up to 7-fold, producing redundant IL-6 and TNFα and activating HSCs though TNFα and/or direct cell contact (26). Rahman AH et al. proposed that DCs regulate the number and activity of cells (e.g. natural killer (NK) cells and CD8+ cells) which involved in fibrosis progression and tissue remodeling (27).

We found that NASH-induced genes in cells from the MMD system were highly enriched for the pathways responsible for fatty acid catabolism (adipor1), extracellular matrix (ECM) remodeling (Rac1, TGFβ1) and immunoregulation (Jun, Socs3, SPP1). By IHC, the expression levels of SPP1, Jun, Socs3 and Rac1 in NASH mice with fibrosis were increased significantly compared with those in control mice. Previous studies have shown that the expression of SPP1 was positively correlated with liver steatosis, inflammation, fibrosis and insulin resistance in obese individuals or mice (28, 29). Multiple signaling pathways, such as the extrinsic apoptosis and fibroblast proliferation, which medicated by SPP1 could regulate the progression of NASH (30). As we all known, c-Jun is an immediate early gene that is regulated by the N-terminal kinase of c-Jun (JNK) (31). It was reported that the expression level of c-Jun elevated with the progression from liver steatosis to NASH (32). In human adipocytes, JNK2 was proven to be involved in fatty acid synthesis through regulating SREBP-1c (33). Rac1, the small GTP-binding protein, is required for saturated fatty acids (SFA)-stimulated MLK3-dependent JNK activation in hepatocytes (34). SOCS3, as a suppressor of cytokine signaling, is considered to promote insulin resistance by inhibiting insulin and leptin signaling during the inflammatory response (35). However, the relationship between the expression of these genes and NASH fibrosis was not clear. In this study, we proposed for the first time that they are related to fibrosis in NASH (**Supplementary Figure 8**).

During our research, Fmnl1 was upregulated in the terminal path of the transition trajectory, and its expression was positively associated with collagen deposition. Fmnl1 is an essential component of macrophage podosomes (36). In the liver, moderate to strong staining of Fmnl1 was shown in macrophages, whereas hepatocytes and biliary epithelium remained negative (37, 38). The expression level of Fmnl1 was associated with cell phagocytosis, adhesion and podosome dynamics, migration and survival of macrophages (37, 39–42). Targeted suppression of Fmnl1 resulted in decreases in macrophage adhesion and migration (36, 42). Additionally, the expression of Fmnl1 was specifically upregulated during monocyte differentiation to macrophages (36). Therefore, combined the results of this study, we speculated that the mechanism of Fmnl1 participating in NASH fibrosis might be that the increased expression of Fmnl1 facilitated the differentiation of monocytes to macrophages and the dynamic changes of podosomes, promoted the release of pro-fibrogenic cytokines and activated HSCs, thereby promoted the production of liver collagen in NASH.

Myh9 was also upregulated in the terminal path of the transition trajectory, and its expression was positively associated with NASH fibrosis. Myh9, the gene which encodes the heavy chain (MHCII) of non-muscle myosin II A (NMII-A), is involved in cell migration, adhesion, division, polarity and morphogenesis and signal transduction (43, 44). Previous studies have suggested that Myh9 was highly upregulated in the process of neutrophil differentiation and played a necessary and fundamental role in neutrophil trafficking (45–47). Furthermore, in hepatocellular carcinoma (HCC), targeting Myh9 improved the survival of HCC-bearing mice markedly and promoted sorafenib sensitivity of HCC cells (48). In our study, we proposed for the first time that Myh9 was associated with liver fibrosis in NASH; however, the mechanism was unclear. It is possible that Myh9 participates in the differentiation and migration of monocytes and then communicates with HSCs to promote collagen formation.

Although our study used scRNA-seq data and our own samples to verify the correlation between characteristic genes in MMD system and the progression of NASH fibrosis, there were some limitations to be considered. First, the mechanism of Fmnl1 and Myh9 genes which involved in NASH fibrosis was still unclear. In the future, we will try our best to explore the signaling pathway of these two genes in collagen production. Second, due to the limitation of our own liver samples, the mechanism of the interaction between Fmnl1 and Myh9 in the NASH progression has not been fully clarified. Third, in this research, only cross-sectional samples were used for the validation of Fmnl1 and Myh9 expression. It was necessary for us to include cohort samples of NASH fibrosis to deeply analyze the clinical value of the characteristic genes in MMD systems.

## 5 Conclusions

During NASH, monocytes, macrophages and DCs in the MMD system are diverse and involved in fibrogenesis. The expression of Fmnl1 and Myh9 in the transition trajectory of the MMD system was related to liver collagen deposition and fibrosis progression in NASH.

## Data availability statement

The datasets presented in this study can be found in online repositories. The names of the repository/repositories and accession number(s) can be found in the article/**Supplementary Material**.

## Ethics statement

This study was approved by the Ethics Committee of Peking University People’s Hospital (2022PHB088-001). The patients/participants provided their written informed consent to participate in this study. This study was approved by the Ethics Committee of Peking University People’s Hospital (2021PHE111).

## Author contributions

XW, HR, and FL contributed to conception and design of the study. ZW, BL, RJ, and YS constructed mouses models. RF, XC, RH, XL, and JY performed the experiments. XW, LW, HR, and FL analyzed and interpreted of data. XW and FL wrote the first draft of the manuscript. FL and HR made critical revision of the manuscript for important intellectual content. All authors contributed to manuscript revision, read, and approved the submitted version.
