# Supplementary material for: Characteristic gene expression in the liver monocyte-macrophage-DC system is associated with the progression of fibrosis in NASH
Source: Front Immunol. 2023 Feb 24;14:1098056. doi: 10.3389/fimmu.2023.1098056 (PMC9998489; doi:10.3389/fimmu.2023.1098056)
Supplement: Supplementary file 1 [file DataSheet_1.pdf]

Supplementary Material

1 Supplementary Data

The datasets in this study can be found in online repositories. The name of the repository is GEO database. This data can be found here: [https://www.ncbi.nlm.nih.gov/geo/query/acc.cgi?acc=GSE216836], accession number: GSE216836.

2 Supplementary Figures

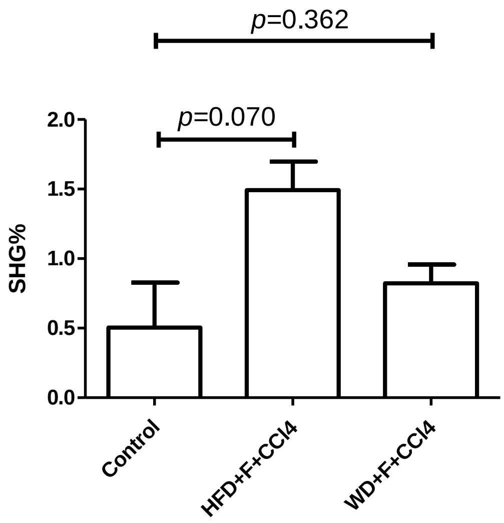

Supplementary Figure 1. The collagen deposition level in control and NASH mice.

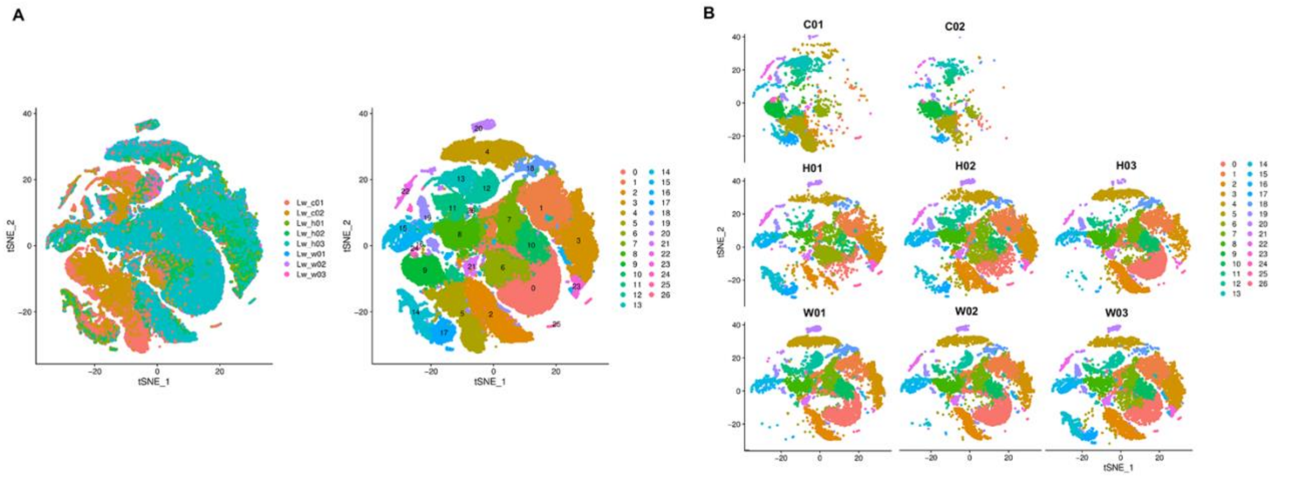

Supplementary Figure 2. ScRNA-seq profiling of liver cells from normal and NASH mice with fibrosis, related to Figure 1. (A-B) T-distributed stochastic neighbor embedding (t-SNE) plot of 27 cell clusters from 2 control mice and 8 NASH mice with fibrosis. ps: C01-02: control mice 01-02; H01-03: HFD+F+CCl4 mice 01-03; W01-03: WFD+F+CCl4 mice 01-03.

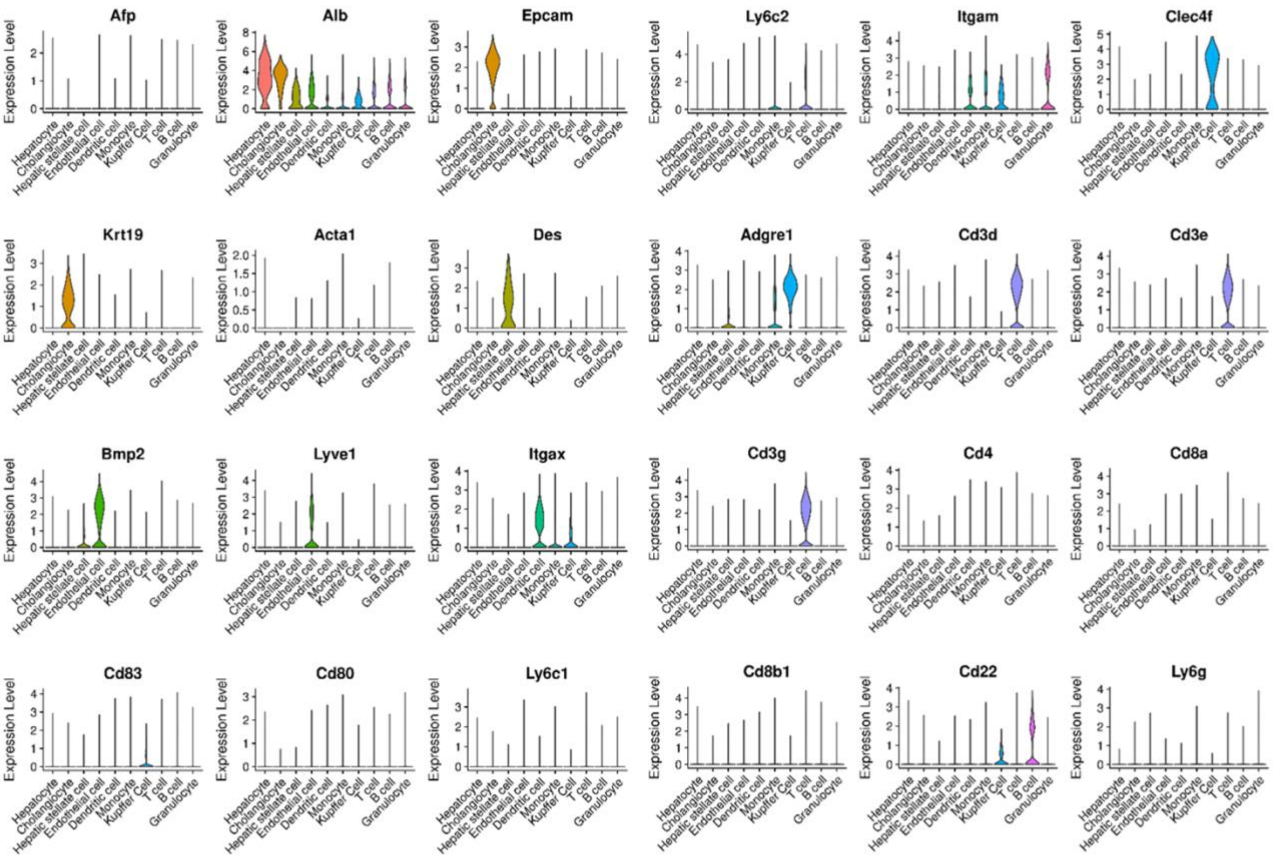

Supplementary Figure 3. Violin plot showing the expression levels of marker genes defined for all cell types.

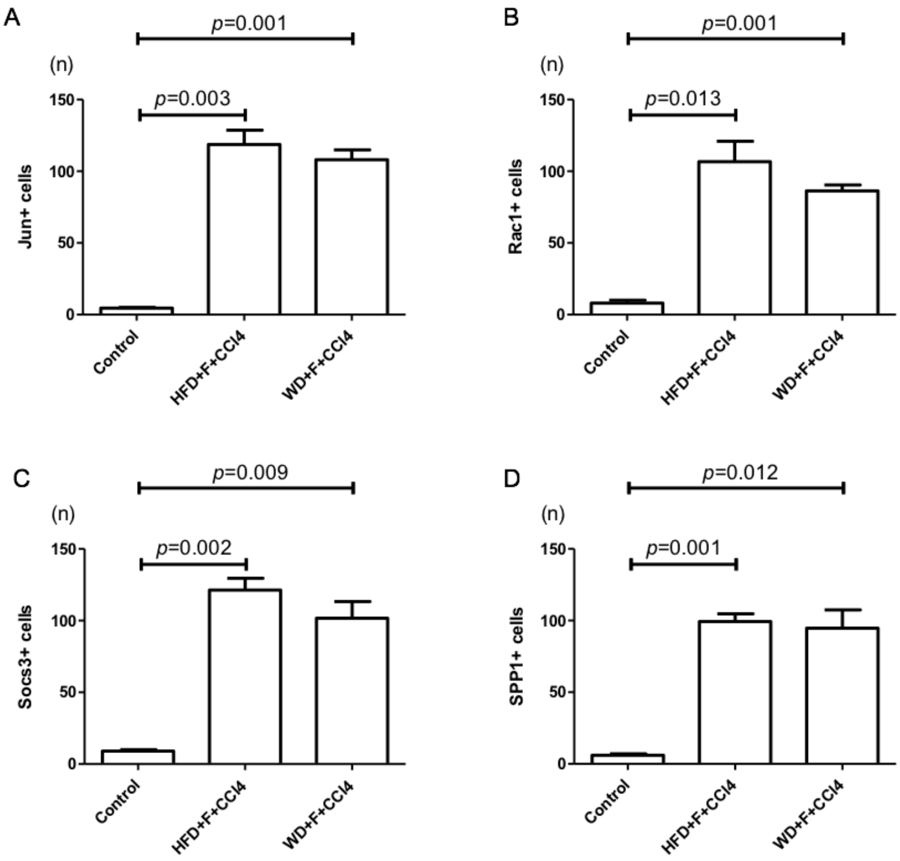

Supplementary Figure 4. The numbers of (A) Jun^{+} cells, (B) $Rac1^{+}$ cells, (C) $Socs3^{+}$ cells and (D) $SPP1^{+}$ cells in control and NASH mice.

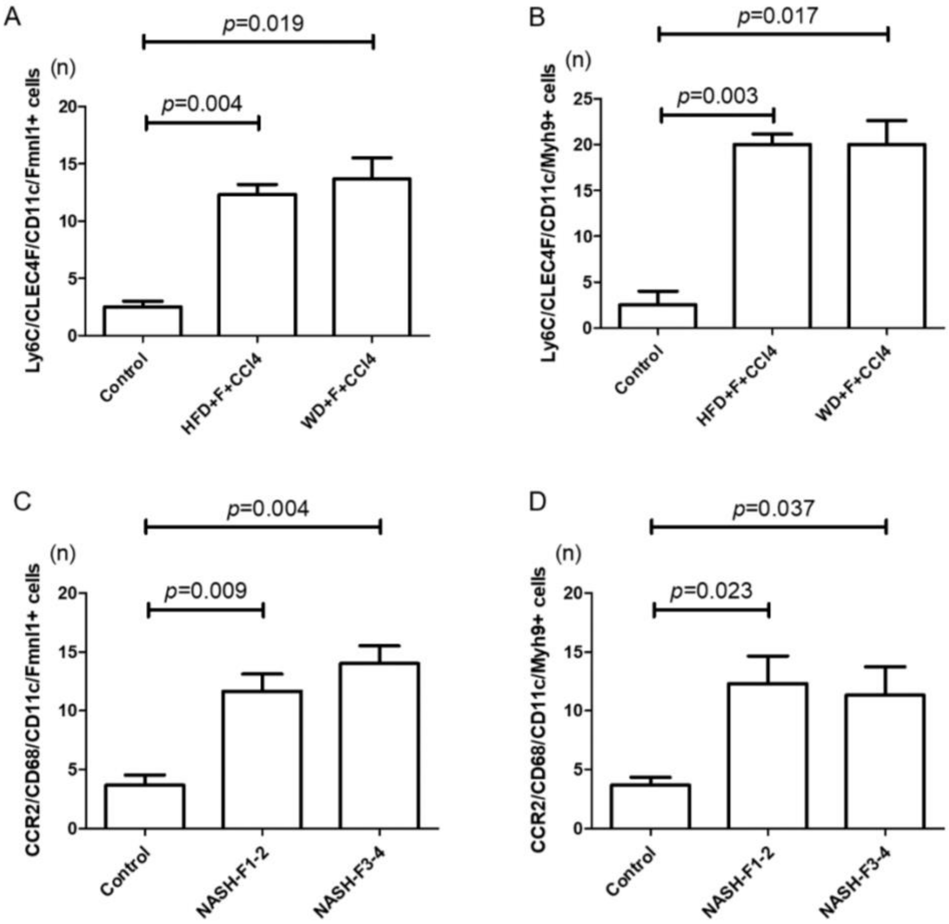

Supplementary figure 5. The expression level of Fmnl1 and Myh9 in cells of MMD system form NASH mice and NASH patients. ps: F1-2: Fibrosis stage 1-2, F3-4: Fibrosis stage 3-4.

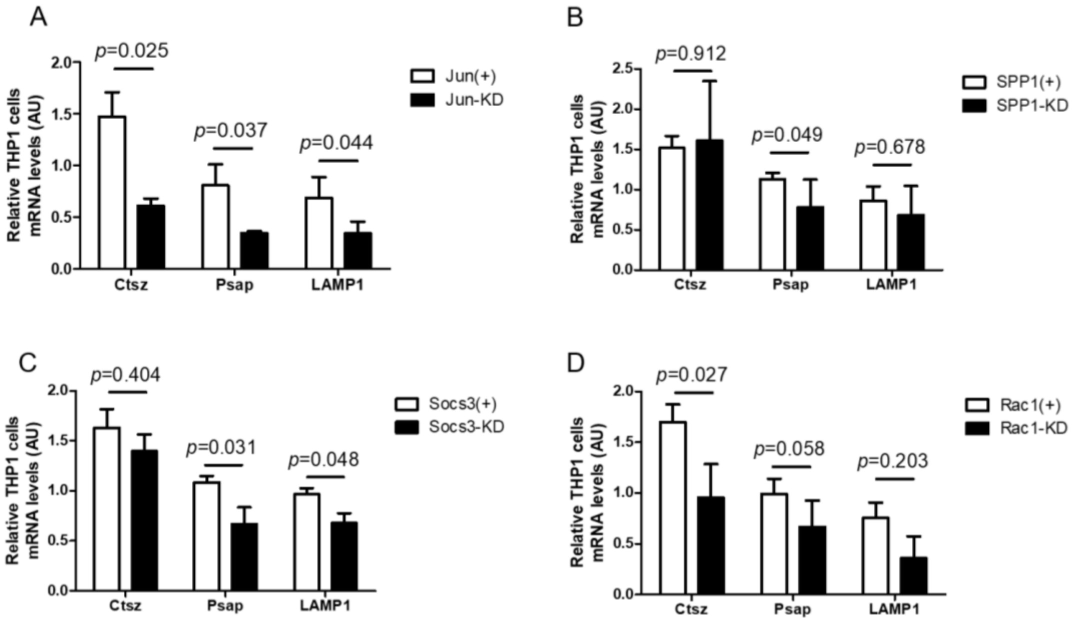

Supplementary figure 6. The relative mRNA levels of Ctsz, Psap and LAMP1 in PA treated THP1 cells after (A) Jun, (B) SPP1, (C) Socs3 or (D) Rac1 was knock-down. Ps: PA, palmitic acid.

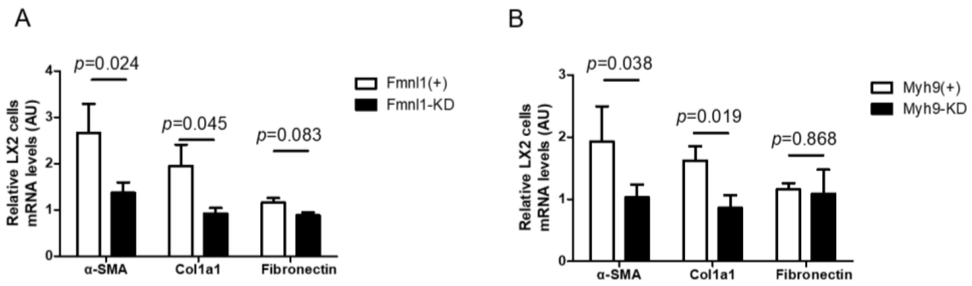

Supplementary figure 7 After (A) Fmnl1 or (B) Myh9 was knock-down (KD) in THP1 cells, the relative mRNA levels of α-SMA, Col1a1 and Fibronectin in LX2 cells from PA treated THP1 and LX2 cocultures.

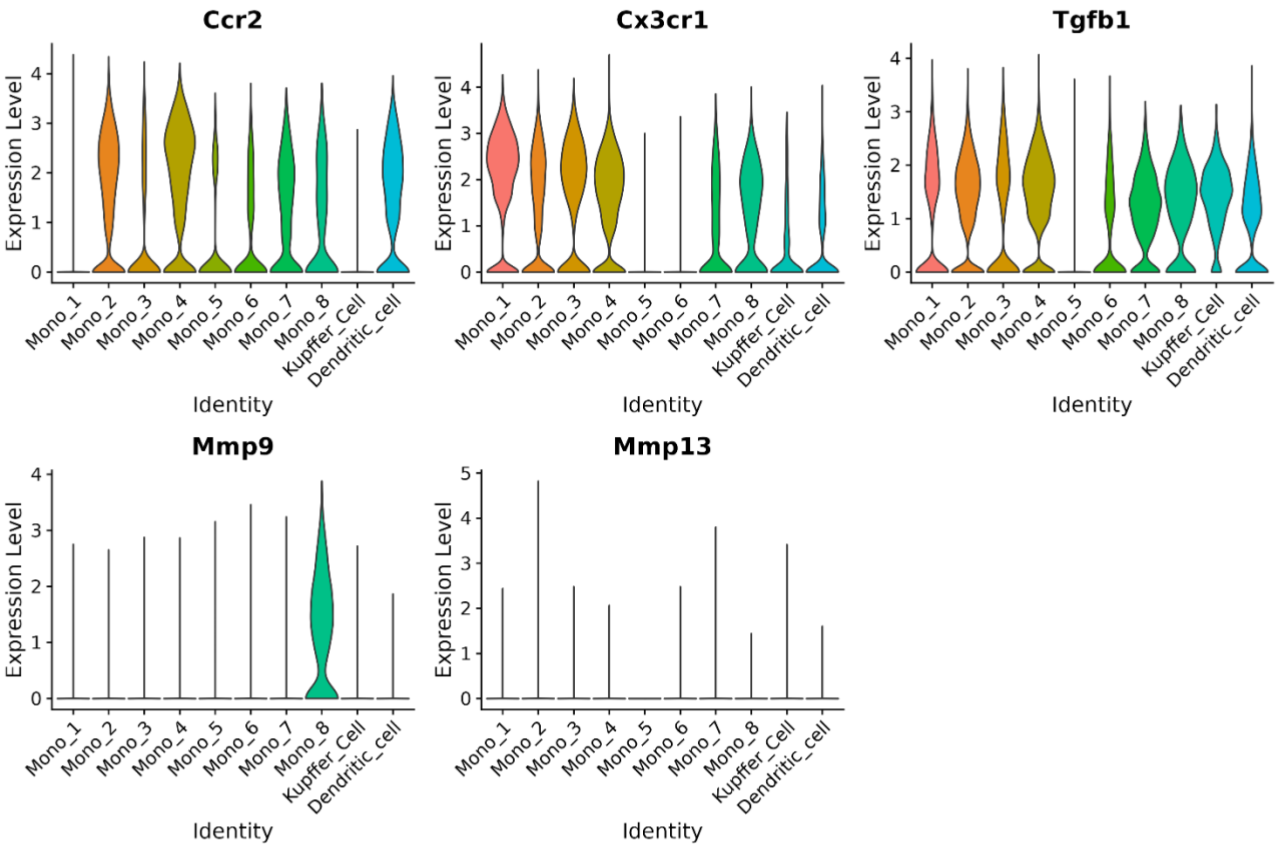

Supplementary Figure 8. The expression level of CCR2, CX3CR1, TGFβ1, MMP9 and MMP13 in different cells of monocyte-Kupffer cell-DC (MMD) system.

3 Supplementary Tables

Supplementary Table 1 The siRNA sequences of representative molecules

Gene name	siRNA sense (5'-3')	siRNA antisense (5'-3')
Human-Jun	ACGCAAACCUCAGCAACUUTT	AAGUUGCUGAGGUUUGCGUTT
Human-Spp1	CGACUCUGAUGAUGUAGAUTT	AUCUACAUCAUCAGAGUCGTT
Human-Socs3	UCAAGCUGGUGCACCACUATT	UAGUGGUGCACCAGCUUGATT
Human-Rac1	CUGGACAAGAAGAUUAUGATT	UCAUAAUCUUCUUGUCCAGTT
Human-Fmnl1	CGUGCGUGCUCAUGAUAAATT	AAUGUGGUUGGUCCUCAGGTT
Human-Myh9	CCUGAGGACCAACCACAUUTT	UUUAUCAUGAGCACGCACGTT
